# Conformational changes and translocation of tissue-transglutaminase to the plasma membranes: role in cancer cell migration

**DOI:** 10.1186/1471-2407-14-256

**Published:** 2014-04-11

**Authors:** Ambrish Kumar, Jianjun Hu, Holly A LaVoie, Kenneth B Walsh, Donald J DiPette, Ugra S Singh

**Affiliations:** 1 Department of Pathology, Microbiology and Immunology, School of Medicine, University of South Carolina, Columbia, SC 29209, USA; 2 Department of Computer Science and Engineering, University of South Carolina, Columbia, SC, USA; 3 Department of Cell Biology and Anatomy, School of Medicine, University of South Carolina, Columbia, SC, USA; 4 Department of Pharmacology, Physiology and Neuroscience, School of Medicine, University of South Carolina, Columbia, SC, USA; 5 Department of Internal Medicine, School of Medicine, University of South Carolina, Columbia, SC, USA

**Keywords:** Cell migration, Invasion, Neuroblastoma, Resveratrol, Tissue-transglutaminase

## Abstract

**Background:**

Tissue-transglutaminase (TG2), a dual function G-protein, plays key roles in cell differentiation and migration. In our previous studies we reported the mechanism of TG2-induced cell differentiation. In present study, we explored the mechanism of how TG2 may be involved in cell migration.

**Methods:**

To study the mechanism of TG2-mediated cell migration, we used neuroblastoma cells (SH-SY5Y) which do not express TG2, neuroblastoma cells expressing exogenous TG2 (SHY_TG2_), and pancreatic cancer cells which express high levels of endogenous TG2. Resveratrol, a natural compound previously shown to inhibit neuroblastoma and pancreatic cancer in the animal models, was utilized to investigate the role of TG2 in cancer cell migration. Immunofluorescence assays were employed to detect expression and intracellular localization of TG2, and calcium levels in the migrating cells. Native gel electrophoresis was performed to analyze resveratrol-induced cellular distribution and conformational states of TG2 in migrating cells. Data are presented as the mean and standard deviation of at least 3 independent experiments. Comparisons were made among groups using one-way ANOVA followed by Tukey-Kramer ad hoc test.

**Results:**

TG2 containing cells (SHY_TG2_ and pancreatic cancer cells) exhibit increased cell migration and invasion in collagen-coated and matrigel-coated transwell plate assays, respectively. Resveratrol (1 μM-10 μM) prevented migration of TG2-expressing cells. During the course of migration, resveratrol increased the immunoreactivity of TG2 without affecting the total TG2 protein level in migrating cells. In these cells, resveratrol increased calcium levels, and depletion of intracellular calcium by a calcium chelator, BAPTA, attenuated resveratrol-enhanced TG2 immunoreactivity. In native-polyacrylamide gels, we detected an additional TG2 protein band with slower migration in total cell lysates of resveratrol treated cells. This TG2 form is non-phosphorylated, exclusively present in plasma membrane fractions and sensitive to intracellular Ca^2+^ concentration suggesting a calcium requirement in TG2-regulated cell migration.

**Conclusions:**

Taken together, we conclude that resveratrol induces conformational changes in TG2, and that Ca^2+^-mediated TG2 association with the plasma membrane is responsible for the inhibitory effects of resveratrol on cell migration.

## Background

Tissue-transglutaminase (TG2) possesses Ca^2+^-dependent transamidation activity by which it catalyzes the cross-linking of proteins via the formation of proteolytically resistant ϵ-(γ-glutamyl) lysine isopeptide bonds between the glutamine residue of one protein and the lysine residue of another protein, polyamine, or monoamine [1-3]. In addition, TG2 acts as a G-protein with GTP-binding and hydrolyzing activity which regulates transamidation function [4,5]. In the extracellular matrix (ECM), TG2 mediates cell-ECM interactions through fibronectin and integrins [6,7] and promotes cell attachment, migration and invasion [8,9]. When TG2 is present at the plasma membrane, it acts as a G-protein and participates in α1-adrenergic signaling [4].

Depending upon the cell type and cellular distribution, TG2 is physiologically involved in cell differentiation, cell death, migration, invasion or survival [10,11]. The increased cellular level of TG2 contributes to the development of drug resistance and metastatic phenotype in several cancer cell types such as that of pancreatic cancer [12], ovarian cancer cells [13,14], breast cancer [15], glioblastoma and malignant melanoma [16]. Studies carried out in an ovarian cancer xenograft model [17] and in mammary epithelial cell lines [18] indicated a direct correlation of TG2 with cancer cell invasion and tumor metastasis. The suppression of TG2 expression, using a TG2 inhibitor or siRNA, induces apoptosis in pancreatic cancer tissue [19]. Moreover, increased level of TG2 by epidermal growth factor (EGF) treatment protects cancer cells from doxorubicin-induced apoptosis highlighting the role of TG2 in cancer cell survival [20]. In our previous studies we reported that TG2 plays a key role in retinoic acid-induced cell differentiation [21,22]. However, the mechanism underlying tissue-transglutaminase mediated cell migration is not well known.

In the present study, using neuroblastoma wild type SH-SY5Y cells, SH-SY5Y cells with overexpressing TG2 (SHY_TG2_) and pancreatic cancer cells (which express higher basal TG2), we have demonstrated that TG2 promotes both cell migration and invasion. To address the molecular mechanism(s) involved in TG2-mediated cancer cell migration, we used a polyphenolic compound, resveratrol (3,5,4’-trihydroxystilbene) which is found in red grapes and blueberries and has shown promising results in the treatment of neuroblastoma and other cancers. However, high concentrations of resveratrol were used in these studies and were focused on resveratrol-induced cell death and the underlying signaling mechanisms [23-27]. The goal of our study was to determine if resveratrol at lower concentrations affects cancer cell migration and invasion and whether it targets TG2 to modify endpoints.

Our data suggest that resveratrol reduces the migration of TG2-containing cells (SHY_TG2_ and pancreatic cancer cells). In migratory cells, resveratrol increases Ca^2+^ levels and converts TG2 into open extended form (as detected by non-denaturing gel electrophoresis). The conversion of TG2 is sensitive to Ca^2+^ levels, and the open extended form is exclusively present in the plasma membrane fraction of migratory SHY_TG2_ and pancreatic cancer cells. Together, we show that Ca^2+^-mediated TG2 association to the cell membrane is responsible for the inhibitory effects of resveratrol on cell migration.

## Methods

### Cell culture

Human neuroblastoma SH-SY5Y cells (ATCC, Manassas, VA), SHY_vector_, SHY_TG2_, and SHY_mutant_ cells were maintained in Dulbecco’s modified Eagle’s medium (DMEM) with 10% fetal bovine serum (FBS; Atlanta Biologicals, Lawrenceville, GA). The pancreatic cancer cell lines Panc-28 and Hs766T were maintained in RPMI medium with 10% FBS [12].

### Scratch assays for cell migration

The cell mobility was determined by scratch assays [28]. Briefly, cells were grown in complete culture medium (with 10% FBS) to a confluent monolayer (up to 90%) in 100 mm^2^ tissue culture dishes, and treated with 10 μM mitomycin c (Sigma-Aldrich, St. Louis, MO) for 30 min to inhibit cell proliferation. Cells were washed twice with complete medium and incubated with 1 μM or 10 μM of resveratrol (Sigma-Aldrich, St. Louis, MO). After 24 h incubation, a wound was made by scratching the dish uniformly with a pipette tip, washed twice with complete culture medium, and cells were further grown in complete culture medium in the presence of respective concentrations of resveratrol. Cells treated with a similar volume of dimethyl sulfoxide (DMSO) were used as control. Cell migration into the empty space was monitored by capturing images at different time points. The distance covered by cells in the empty area was calculated in %.

### Collagen-transwell cell migration and matrigel-transwell cell invasion assays

Cell migration studies were performed using transwell inserts with a filter of 8 μm pore size (BD bioscience, San Jose, CA) and coated with 10 μg/ml collagen IV (Santa Cruz Biotechnology, Santa Cruz, CA). After 48 h treatment with resveratrol or DMSO, cells were trypsinized and 2 × 10^4^ cells in serum-free medium with resveratrol were seeded into the upper chamber of the transwell insert. Culture medium with 10% FBS was added to the lower chamber. After 15 h incubation, cells migrated on the lower surface of inserts were stained with Hema3 stain (Fisher Scientific, Kalamazoo, MI), photographed, and counted from 10 randomly selected fields. Cell invasion studies were performed using matrigel-coated transwell plates (BD bioscience, San Jose, CA) following experimental procedure similar to collagen-transwell migration assays.

### Immunofluorescence microscopy

Scratch assays as described above were performed in Lab-TekII chamber slides (Fisher Scientific, Pittsburgh, PA). Cells were fixed with 4% paraformaldehyde in phosphate buffered saline (PBS), permeabilized with 0.2% Triton X-100/PBS, blocked with 5% bovine serum albumin (BSA) in PBS with 0.1% Tween-20 (PBST) at 4°C for overnight, and incubated with monoclonal anti-TG2 antibody (Neomarker/Thermoscientific, Rockford, IL) in 5% BSA/PBST at 4°C for overnight. Primary antibodies were detected with rhodamine-labeled anti-mouse IgG antibody (2 h incubation at room temperature). After washing with PBST, slides were mounted with Vectashield mounting media (Vector Laboratories, Burlingame, CA), and visualized under Nikon E-600 fluorescence microscope [29]. Nuclei were counterstained with DAPI (Sigma). TG2 on the outer surface of plasma membrane was detected as described by Akimov and Belkin [8]. Briefly, cells grown in chamber slides were incubated with anti-TG2 antibody (10 μg/ml in PBS) at 37°C for 4 h, fixed with 4% paraformaldehyde/PBS for 20 min, and blocked with 5% BSA/PBS for 2 h. Primary antibody (anti-TG2) was detected by rhodamine-conjugated goat anti-mouse IgG antibody under Nikon E-600 fluorescence microscope.

### Isolation of total cell proteins, plasma membrane proteins and Western blotting

After treatments in scratch assays, cells near (~1 mm from the scratch) and away from the scratch were collected separately. For total cell extract preparation, cells were lysed with cell lysis buffer (Cell Signaling Technology, Danvers, MA), incubated for 30 min at 4°C, centrifuged at 12,000 g for 10 min at 4°C and supernatant (containing total cell proteins) was collected. Cytoplasmic and plasma membrane protein enriched fractions were prepared using plasma membrane protein extraction kit (BioVision Inc, Milpitas, CA). Extracted proteins were stored at −80°C, and protein quantitation was performed by bicinconinic acid method (Pierce/Thermo-Scientific, Rockford, IL). Proteins were separated by SDS-polyacrylamide gel electrophoresis followed by Western blots as described previously [30]. The primary antibodies used were: TG2 (Neomarker/Thermoscientific), glyceraldehyde 3-phosphate dehydrogenase (GAPDH), Na^+^/K^+^-ATPase (Santa Cruz Biotechnology, Santa Cruz, CA), and p-tyrosine (Millipore, Billerica, MA).

For total native protein isolation, cells were lysed in lysis buffer (0.1 M Tris, pH 7.4, 0.05% Triton X-100, 0.5 mM PMSF, 10 μg/ml leupeptin, 10 μg/ml pepstatin, 1 mM NaF, 1 mM Na_3_VO_4_, 1 mM β − glycerophosphate, and 2.5 mM sodium pyrophosphate) for 1 h on ice. After centrifugation at 12,000 g for 15 min at 4°C, the supernatant (containing native proteins) was collected, and quickly stored at −80°C until use. For non-denaturing gel electrophoresis, equal amount of proteins (5 μg) were mixed with 5× loading buffer (without reducing agents and SDS), and separated in 12% native gel (without SDS) in running buffer (25 mM Tris, 192 mM glycine) for 12 h at 4°C. Separated proteins were transferred to PVDF membrane and Western blots were performed as described above.

### Transglutaminase activity and GTP-binding assays

Extracted proteins (5 μg) from total cells and cytoplasmic or membrane fractions were used to detect the transglutaminase activity of TG2 using tissue-transglutaminase colorimetric microassay kit (Covalab, Villeurbanne, France). The assay uses biotinylated T26 peptide (Biotin-pepT26) and spermine as substrates. In the presence of TG2, spermine is incorporated into the γ carboxamide of the glutaminyl residue of the biotin-pepT26 to form a biotin-pepT26-γ-glutamyl spermine. In brief, protein samples with assay mixture containing Biotin-pepT26 were incubated in spermine-coated 96-well microtiter plate, and the incorporation of spermine to Biotin-pepT26 was detected by sptreptavidin-avidine labelled peroxidase system. The color intensity as an indicator of TG2 activity was measured at 450 nm in a Spectramax spectrophotometer (Molecular Devices, Sunnyvale, CA). Photoaffinity labeling of GTP binding proteins with [α^32^P]-GTP was performed as described previously [31]. Briefly, 5 μg protein samples (in 20 μl) in 96 well-plates were incubated with 20 μl of reaction buffer (50 mM Hepes, pH 8.0, 1 mM EDTA, pH 8.0, 1 mM DTT, 10% glycerol) containing 2 μCi of [α^32^P]-GTP for 30 min at room temperature. Reactions were irradiated with UV-light (254 nm) for 15 min on ice. After irradiation, samples were mixed with 5× SDS loading buffer, boiled for 5 min and separated on SDS-polyacrylamide gels. Protein samples were transferred to PVDF membrane and the membranes were exposed to x-ray film. Similar membranes were used for Western blot and probed with anti-TG2 antibody.

### Detection of Ca^2+^ levels by fluorescence microscopy

The level of Ca^2+^ in cells was determined by fluorescence microscopy using a membrane permeable Ca^2+^ indicator dye, Fluo4-AM (Molecular Probes, Grand Island, NY). Briefly, after treatment with resveratrol in scratch assays in Lab-TekII chamber slides, cells were incubated with Tyrode’s solution (20 mM Hepes, pH 7.4, 130 mM NaCl, 4.7 mM KCl, 1 mM MgSO_4_, 1.2 mM KH_2_PO_4_, 5 mM glucose) containing 10 μM of Fluo4-AM dye and 0.1% pluronic F-127 (Molecular Probes) for 1 h at 37°C in a 5% CO_2_ incubator. After washing twice with Tyrode’s solution, cells were further incubated for 30 min at room temperature in the dark for de-esterification of dye. Slides were mounted in Vectashield mounting media (Vector Laboratories), and were visualized under fluorescence microscope. Fluorescence intensity in cells present near the scratch was quantified using Image-Pro Plus software and plotted.

### Data analyses

Data are presented as the mean and standard deviation of at least 3 independent experiments. Comparisons were made among groups using one-way ANOVA followed by Tukey-Kramer ad hoc test (GraphPad software, La Jolla, CA). A *p*-value < 0.05 was considered significant.

## Results

### TG2 enhances the migration and invasion of cancer cells which is inhibited by resveratrol

**Figure 1:**
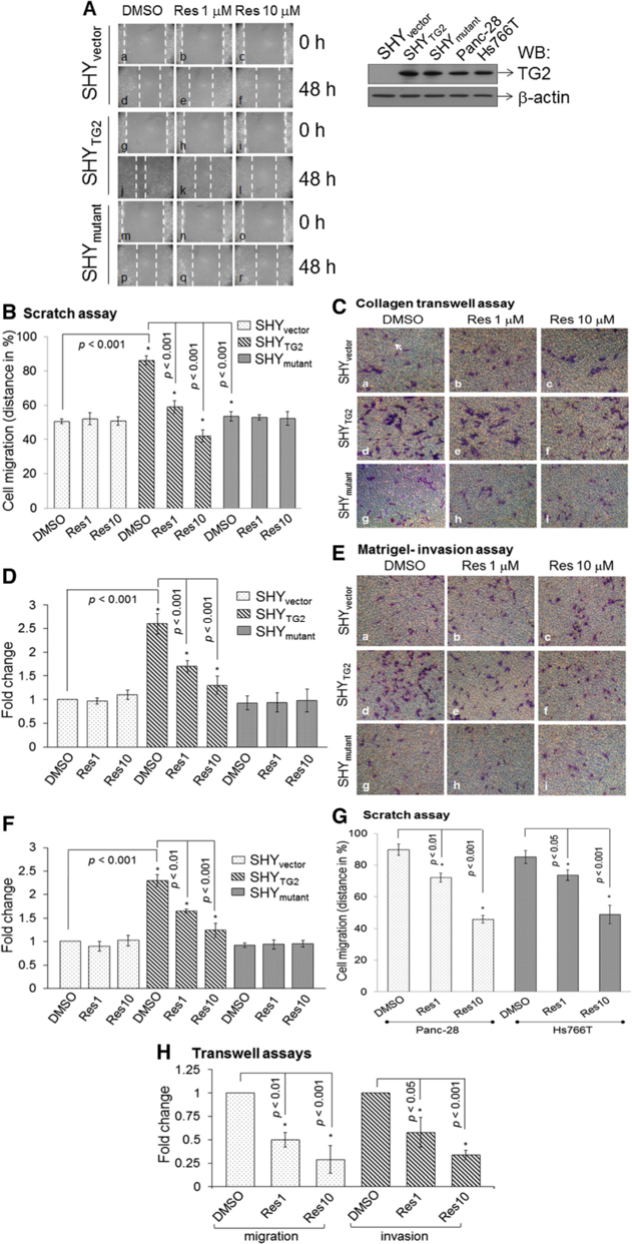
**Cell migration and invasion assays. (A)** Representative images from the scratch assays showing the migration of SHY_vector_, SHY_TG2_, and SHY_mutant_ cells. Mytomycin C treated cells were preincubated with resveratrol (1 μM and 10 μM) for 24 h; a scratch was made and further incubated with resveratrol. At 0 h and 48 h after scratch, cells were photographed. Dotted lines represent the edge of migrated cells in the scratch. Upper right panel: Western blot for TG2 protein using 10 μg total cell extract from SHY_vector_, SHY_TG2_, SHY_mutant_, Panc-28, and Hs766T cells. **(B)** The distance covered by cells in the original empty area was measured and plotted in %. Bars represent mean ± SD of three independent experiments. *p value < 0.05. **(C-E)** Images from collagen-transwell and matrigel-transwell assays. After 48 h with or without resveratrol treatment, cells were trypsinized and seeded on collagen-transwell **(C)** or matrigel-transwell inserts in the presence of resveratrol **(E)**. After 15 h, migrated cells on the lower side of inserts were stained with Hema-3 stain (arrow), counted from ten random fields and plotted **(D and F)**. Bars are mean ± SD of at least three independent experiments and *p value < 0.05. **(G)** Bar diagram represents the migration of Panc-28 and Hs766T cells in scratch assays in the presence of resveratrol as performed with SH-SY5Y cells. Migrated cells into the original empty area were photographed and plotted. Bars are mean ± SD of three independent experiments. *p value < 0.05. **(H)** Migration and invasion assays for Panc-28 cells were carried out as with neuroblastoma cells in transwell inserts. Migrated/invaded cells were counted from 10 random fields and plotted. Bars are mean ± SD of three independent experiments. *p value < 0.05.

We first evaluated the role of TG2 in migration of SH-SY5Y neuroblastoma cells by scratch assays and by collagen-transwell plate assays. Since the basal expression level of TG2 is very low in SH-SY5Y cells (nearly undetectable by Western blot, as shown in Figure 1A), we also used SH-SY5Y cells stably overexpressing TG2 protein (SHY_TG2_) [32]. SH-SY5Y cells transfected with only the backbone plasmid (SHY_vector_) were used as a control. Scratch assays in Figure 1A showed that 48 h after scratch, approximately 86% of the empty area of the original scratch was covered by SHY_TG2_ (Figure 1Aj) in comparison to < 50% migration by SHY_vector_ (Figure 1Ad and Figure 1B). Similarly, compared to SHY_vector_ cells, SHY_TG2_ cells exhibited more than a two-fold higher migration in collagen-transwell migration assays (Figure 1Ca and d and Figure 1D), and invasion through matrigel-coated transwell inserts (Figure 1Ea and d and Figure 1F). These results indicate that TG2 is one of the components required for cell mobility.

To detect whether the transglutaminase activity of TG2 is required for the migration, we performed scratch assays with SH-SY5Y cells stably expressing a full length TG2 mutant protein that lacks transamidation activity due to the mutation at amino acid position 277 (C277S, denoted as SHY_mutant_) [32]. Compared to SHY_TG2_ cell migration (~86% of migration, Figure 1Aj), we observed only 53% SHY_mutant_ cellular migration in scratch assays when grown in similar culture conditions (Figure 1Ap). Also, SHY_mutant_ cells had a lower rate of migration and invasion pattern compared to SHY_TG2_ cells in collagen-transwell (Figure 1Cd and g) and matrigel-transwell assays (Figure 1Ed and g), respectively.

The anticancer properties of resveratrol (res) have been well documented [25]. To test whether TG2 plays a role in the inhibitory effects of resveratrol on cancer cell migration and invasion, we performed migration and invasion assays in the presence of resveratrol. Exposure to resveratrol (1 μM or 10 μM) significantly inhibited the migration of SHY_TG2_ cells in scratch assays (Figure 1Aj, k and l and B) and in collagen-transwell assays (Figure 1Cd, e, f, and D) as well as the invasion through the matrigel-barrier in transwell inserts (Figure 1Ed, e, f and bar in F). In contrast, resveratrol did not affect SHY_vector_ and SHY_mutant_ cell migration (in scratch assays or through collagen-transwell plates, Figure 1A-D) or invasion (through matrigel-transwell inserts, Figure 1E and F).

We further verified the resveratrol-inhibited migration and invasion pattern in TG2 expressing human pancreatic cancer cell lines Panc-28 and Hs766T [12]. After 48 h of DMSO treatment in scratch assays, Panc-28 and Hs766T cells migrated into the empty area by almost 89% and 85%, respectively. The addition of 1 μM or 10 μM resveratrol significantly reduced the mobility of all these cell lines in a dose dependent manner (Figure 1G). Resveratrol (1 or 10 μM) also significantly reduced the migration and invasion of Panc-28 cells in transwell-migration and transwell-invasion assays, respectively (Figure 1H).

### Resveratrol increases the immunoreactivity of TG2 in migratory SHY_TG2_ and pancreatic cancer cells

**Figure 2:**
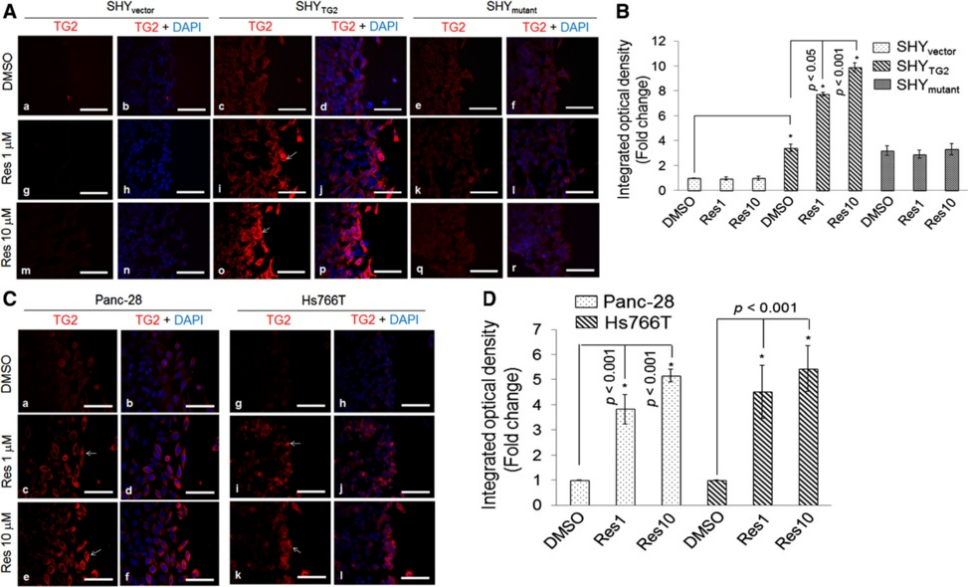
**Immunofluorescence signal for TG2 in SH-SY5Y cells and pancreatic cancer cells.** Representative immunofluorescence images showing TG2 level (red color) in SHY_vector_, SHY_TG2_ and SHY_mutant_ cells **(A)**, and Panc-28, Hs766T cells **(C)**. After treatment with resveratrol (1 or 10 μM) in scratch assays, cells were labeled with anti-TG2 antibody. Nuclei were counter-stained with DAPI (blue) and slides were examined under fluorescence microscope. Pictures were taken randomly from the edge of scratch and are representative of at least 3 independent experiments. Enhanced red fluorescence (white arrows) in cells present near the scratch depicts increased immunoreactivity for TG2, and quantitated by Image-Pro Plus software and plotted. Corresponding bar diagrams represent the fold change in fluorescence intensity (integrated optical density, IOD) **(B and D)**. *p value < 0.05. Scale bar = 100 μm.

To determine whether resveratrol inhibits the migration of SHY_TG2_ and pancreatic cancer cells by altering the expression and/or cellular distribution of TG2, we first performed immuno-fluorescence studies for TG2 in scratch assays. The immunofluorescence images in Figure 2A demonstrated that the immunoreactivity for TG2 remains equally distributed after 48 h DMSO-treated SHY_TG2_ cells (Figure 2Ac). In contrast, following 48 h resveratrol treatment (1 μM or 10 μM) a significant increase in the fluorescent signal for TG2 (red in color and indicated by arrow) was observed in the SHY_TG2_ cells present only near the scratch (Figure 2Ai and o and B). In the case of the SHY_mutant_ cells, the TG2 immunoreactivity remains unchanged in DMSO and resveratrol 1 μM or 10 μM treated cells (Figure 2Ae, k and q and B). TG2 immunoreactivity was not detected in either untreated or resveratrol treated SHY_vector_ cells (Figure 2Aa, g and m and B). Similarly, 48 h incubation with 1 μM or 10 μM concentration of resveratrol increased TG2 immunoreactivity in Panc-28 (Figure 2Ca-f, and D) and Hs766T cells (Figure 2Cg-l, and D) present near the scratch (as shown by arrow).

### TG2, in response to resveratrol, is not localized to extracellular side of plasma membrane or secreted into the medium

**Figure 3:**
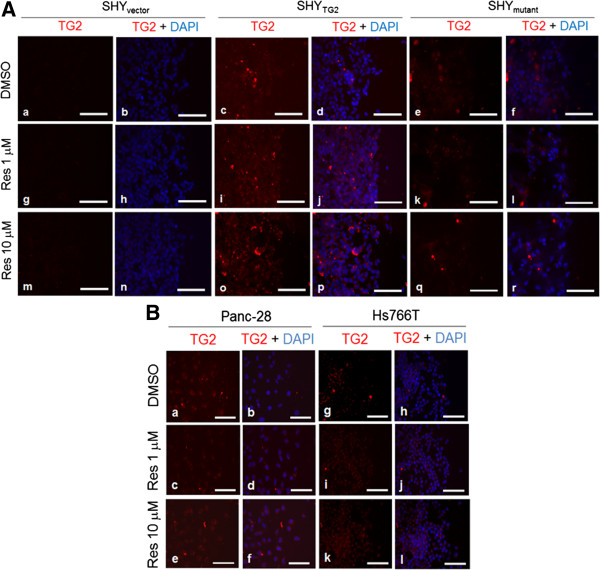
**Determination of extracellular levels of TG2 in response to resveratrol.** Representative immunofluorescence images showing extracellular TG2 level (red) in SHY_vector_, SHY_TG2_, and SHY_mutant_**(A)**, and Panc-28 and Hs766T cells **(B)**. After 48 h with or without resveratrol (1 or 10 μM) treatment in scratch assays, extracellular TG2 was detected using anti-TG2 antibody. Nuclei were stained with DAPI and visualized under fluorescence microscopy. Pictures are representative of at least 3 independent experiments. Scale bar = 100 μm.

To test whether externalization of cytosolic TG2 was involved in the anti-invasive effects of resveratrol, we performed an immunofluorescence assay (to examine TG2 on outer surface of plasma membrane) and ELISA (to detect TG2 levels in culture medium). After 48 h treatment with or without resveratrol in scratch assays, non-permeabilized cells were incubated with anti-TG2 antibody. Our immunofluorescence results in Figure 3A and B demonstrate that resveratrol did not alter the immunoreactivity of TG2 on the external surface of SHY_TG2_ cells (Figure 3Ac, i and o), as well as on Panc-28 (Figure 3Ba-f) and Hs766T cells (Figure 3Bg-l). Similarly, our ELISA result shows that resveratrol did not significantly alter TG2 secretion in culture medium collected from resveratrol-treated and untreated SHY_TG2_ and Panc-28 cells in scratch assays (Additional file 1: Figure S1).

### Resveratrol increases the translocation of TG2 to the plasma membranes of SHY_TG2_ and Panc-28 cells present near the scratch

**Figure 4:**
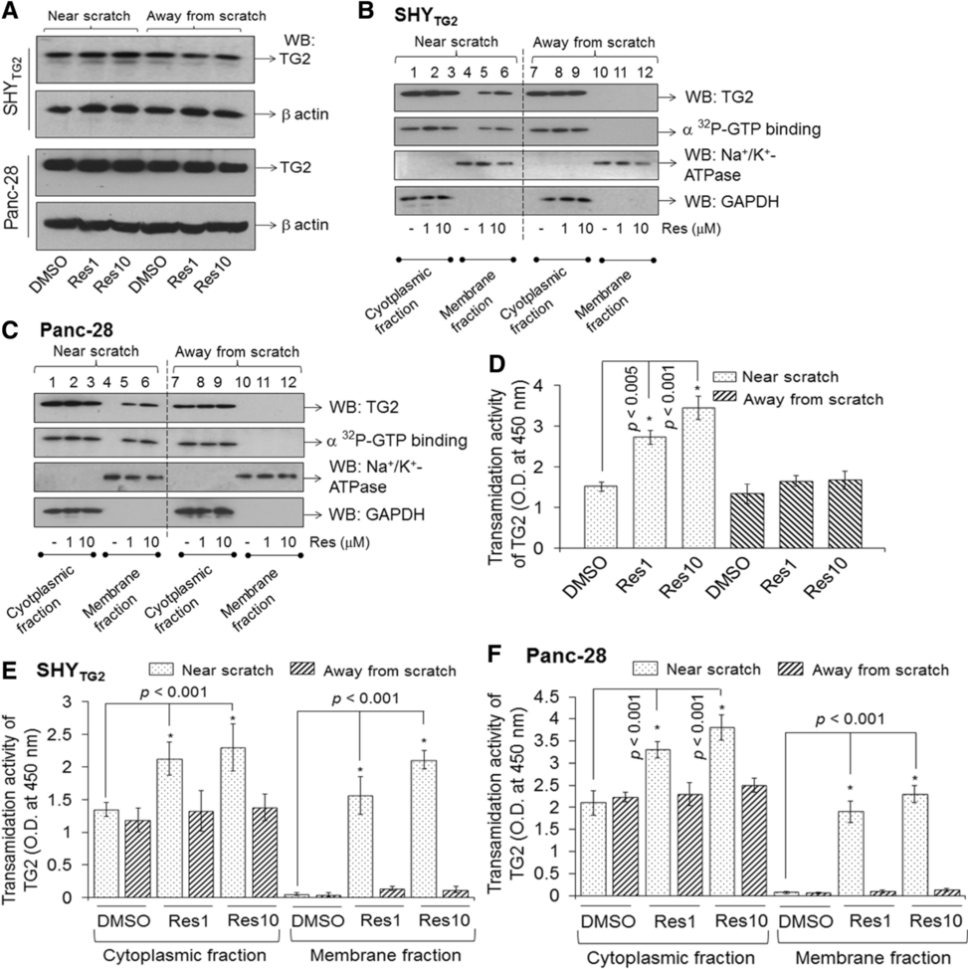
**Cellular distribution and enzymatic activity of TG2 in response to resveratrol. (A)** Representative Western blots showing TG2 protein level in total cell extracts prepared from SHY_TG2_ and Panc-28 cells. After 48 h treatment in scratch assays, cells near the scratch and away from the scratch were collected separately, and total protein extracts were prepared. Equal amount of proteins were separated on SDS-polyacrylamide gels and Western blots for TG2 were performed. Blots were reprobed with β-actin to indicate loading. **(B and C)** GTP-binding and Western blots for TG2 in cytoplasmic and membrane protein fractions prepared from SHY_TG2_**(B)** and Panc-28 cells **(C)**. GTP-binding activity of TG2 was performed by UV-irradiating equal amount of proteins (5 μg) in the presence of [α^32^P]-GTP. UV-irradiated samples were separated by SDS-PAGE, transferred to PVDF membrane and exposed to x-ray films. Similar blots were probed with anti-TG2 antibody for Western blotting. Antibodies against Na^+^/K^+^-ATPase and glyceraldehyde 3-phosphate dehydrogenase (GAPDH) were used as plasma membrane and cytoplasmic protein markers, respectively. **(D, E and F)** Bar diagrams representing transamidation activity of TG2 in SHY_TG2_ and Panc-28 cells. After treatments in scratch assays, total cell lysate of SHY_TG2_**(D)**, and cytoplasmic and membrane protein fractions of SHY_TG2_ and Panc-28 cells **(E and F)** prepared from cells present near and away from scratch, and transamidation activity was measured by colorimetric assay at 450 nm. Experiments were repeated at least three times. *p < 0.05.

To test whether the cellular distribution and/or enzymatic activity of TG2 is altered by resveratrol as it inhibits the migration of SHY_TG2_ and Panc-28 cells, we subsequently determined TG2 protein level and enzymatic activity (GTP-binding as well as transamidation) in these cells. After 48 h in scratch assays, cells near the scratch (~1 mm from the scratch) and away from the scratch were collected separately, total cell extracts were prepared and Western blots with anti-TG2 antibody was performed. Western blots for TG2 in Figure 4A depicted that TG2 protein level is almost similar in total cell extracts prepared from resveratrol-treated or -untreated SHY_TG2_ and Panc-28 cells present near and away from the scratch. However, Western blots in Figure 4B and C (upper panel) demonstrated that TG2 protein band was detected in the membrane fraction of cells present near the scratch only in resveratrol treated SHY_TG2_ and Panc-28 cells, but not in DMSO-treated cells (Figure 4B and C, lanes 4–6). Such a TG2 band was not observed in the membrane fractions from DMSO- or resveratrol-treated cells present away from the scratch (Figure 4B and C, upper panel, lanes 10–12). The cytosolic fractions isolated from close to or away from the scratch had almost similar levels of TG2 proteins in DMSO- and resveratrol-exposed cells (Figure 4B and C, upper panel, lanes 1–3 and 7–9). The fractions that contain TG2 protein exhibit comparable levels of GTP-binding activity (Figure 4B and C, lower panel, lanes 1–3, 5, 6 and 7–9).

The bar diagram in Figure 4D depicted that resveratrol exposure (1 μM or 10 μM) significantly increased the transamidation activity (as determined by tissue transglutaminase colorimetric microassay kit, Covalab) in SHY_TG2_ total cell extracts prepared from the cells present near the scratch (for res 1 μM, *p* < 0.005; and for res 10 μM, *p* < 0.001 vs. DMSO). Such changes in transamidation activity by resveratrol were not observed in SHY_TG2_ total cell extracts prepared from the cells present away from the scratch (Figure 4D). Resveratrol exposure significantly increased transamidation activity of TG2 in cytoplasmic and membrane fractions prepared from SHY_TG2_ and Panc-28 cells present near the scratch, but not in cells present away from the scratch (Figure 4E and F).

### Resveratrol induces conversion of compact TG2 into its open slow mobility form in cells present near the scratch

**Figure 5:**
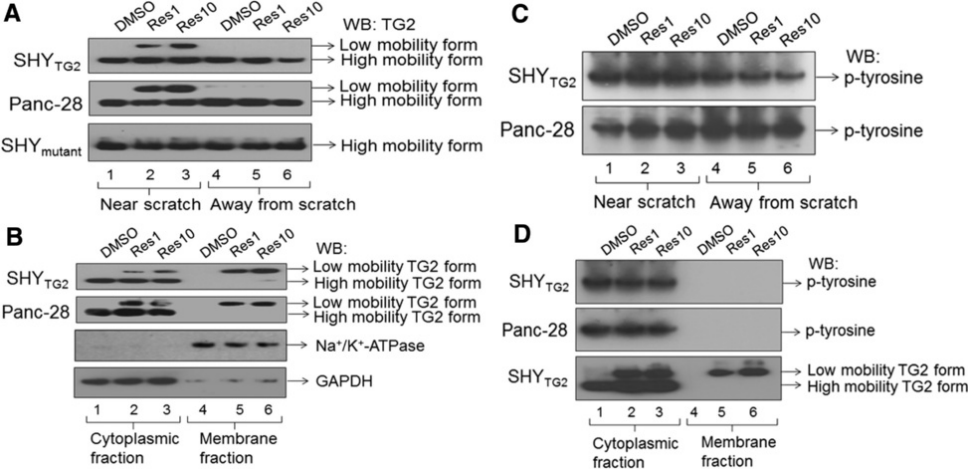
**Detection of TG2 protein under non-denaturing conditions. (A)** Representative Western blots for TG2 in total cellular proteins isolated from resveratrol-treated SHY_TG2_, SHY_mutant_ and Panc-28 cells present near and away from the scratch. Equal amount of proteins were separated on non-denaturing conditions and Western blots were done with anti-TG2 antibody. In addition to the major TG2 band, another TG2 protein band with lower migration was detected in resveratrol-treated SHY_TG2_, and Panc-28 cells present near the scratch. **(B)** Representative Western blots for TG2 with cytoplasmic and membrane protein fractions prepared from DMSO- or resveratrol-treated SHY_TG2_, and Panc-28 cells present near the scratch. Under non-denaturing conditions, TG2 protein band with slower migration was present in both cytoplasmic and membrane protein fractions prepared from resveratrol-treated (1 and 10 μM) cells. **(C and D)** Western blots using anti-tyrosine antibody to detect phosphorylation state of TG2 in total cell extract **(C)** and cytoplasmic and membrane protein fractions **(D)**. Native proteins (100 μg) isolated from resveratrol or DMSO treated SHY_TG2_ and Panc-28 cells present near the scratch, separated on non-denaturing gels followed by Western blot with anti-phosphotyrosine (p-tyrosine). The bottom panel **(D)** represents the Western blot for TG2 in SHY_TG2_ cells.

Next, we sought to determine how resveratrol increases TG2 immunoreactivity only in SHY_TG2_ and pancreatic cancer cells present near the scratch. We subsequently performed Western blots with anti-TG2 antibody under non-denaturing conditions. The representative Western blots in Figure 5A depict that in addition to the major TG2 protein band (~87 kDa) another protein band of slower mobility (~95 kDa) was detected by anti-TG2 antibody in native protein samples from resveratrol-treated SHY_TG2_ and Panc-28 cells present near the scratch (Figure 5A, upper and middle panel, lanes 2 and 3), but not in DMSO-treated cells (Figure 5A, lane 1). However, the slower mobility form of TG2 was not detected in DMSO- or resveratrol-treated SHY_TG2_ and Panc-28 cells present away from the scratch (Figure 5A, lanes 4–6). Despite the appearance of slower mobility TG2 form, we did not detect any change in the level of higher mobility TG2 form. In SHY_mutant_ cells only one TG2 band of higher mobility (~87 kDa) was detected in control and treatment groups (Figure 5A, lower panel, lanes 1–6).

Since resveratrol induces translocation of TG2 to the plasma membranes (Figure 4B and C); we further evaluated the forms of TG2 in membrane fractions. The representative Western blots for TG2 in non-denaturing conditions demonstrated that TG2 protein band with slower migration was only detected in the membrane fractions prepared from resveratrol-treated SHY_TG2_ and Panc-28 cells present near the scratch (Figure 5B, upper two panels, lanes 4–6). A TG2 band of similar mobility was also present in the cytoplasmic fractions prepared from resveratrol-treated cells present near the scratch (Figure 5B, lanes 2 and 3). However, the TG2 protein band with higher mobility was not present in resveratrol-treated membrane protein fractions, but present in the cytoplasmic fraction from DMSO- and resveratrol-treated samples (Figure 5B, lanes 1–6). These data indicated that a resveratrol-induced slower mobility form of TG2 exists in migratory SHY_TG2_ and Panc-28 cells, and this is the exclusive TG2 form present in the plasma membrane of migratory cells.

Further we checked post-translational modification, phosphorylation, of TG2 [33] in native total cellular proteins from SHY_TG2_ and Panc-28 cells present near and away from the scratch, followed by Western blots under non-denaturing conditions using anti-tyrosine antibody. Blots in Figure 5C depict that the anti-tyrosine antibody detects only a single protein band in both resveratrol-treated and untreated native protein samples that corresponds to the TG2 protein of the higher mobility form (lanes 2 and 3). Also, irrespective of resveratrol treatment, anti-tyrosine antibody detects only a single band in native proteins from cytoplasmic fractions, but not in plasma membrane fractions from SHY_TG2_ and Panc-28 cells present near the scratch (Figure 5D, upper two panels). However, we did not observe serine phosphorylation in these conditions (data not shown). This indicates that the higher mobility form of TG2 is the phosphorylated form, and the slower mobility form of TG2, which appears in the presence of resveratrol, is a non-phosphorylated form. This also suggests that resveratrol-induced dephosphorylation of the higher mobility form of TG2 may be involved in the conversion of the compact higher mobility form into slower mobility open structure TG2 form.

### Resveratrol increases intracellular Ca^2+^ levels in cells present near the scratch

**Figure 6:**
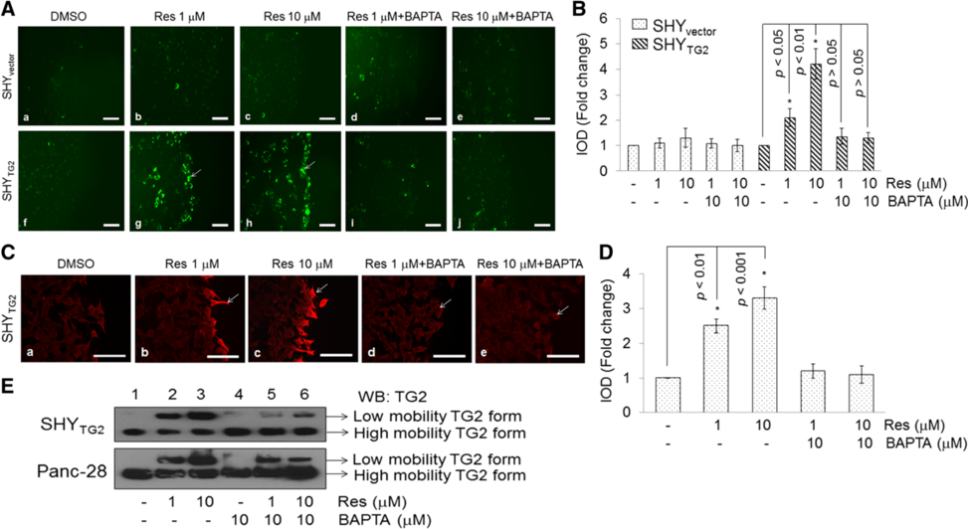
**Changes in intracellular Ca**^**2+ **^**levels in response to resveratrol. (A)** Representative fluorescence images showing the level of intracellular Ca^2+^ in SHY_vector_ and SHY_TG2_ cells. After 48 h resveratrol treatment in scratch assays, cells were labeled with Ca^2+^-indicator dye, Fluo4-AM, for 1 h, and examined under fluorescence microscope using FITC filter. Cells treated with resveratrol were also treated with cell permeable Ca^2+^ chelator, BAPTA-AM (10 μM) to chelate intracellular Ca^2+^ in cells. Scale bar = 100 μm. Fluorescence intensity in cells present near the scratch was measured using Image-Pro Plus software and plotted as fold change in IOD **(B)**. **(C)** Representative immunofluorescence images for TG2 in scratch assays. SHY_TG2_ cells were grown in chamber slides and scratch assays were performed in presence of Ca^2+^ chelator, BAPTA-AM (10 μM), alone or in combination with resveratrol (1 or 10 μM). After 48 h treatments, cells were labeled with anti-TG2 antibody (red) and images were taken randomly from the edge of the scratch using fluorescence microscope equipped with rhodamine filter. Enhanced red intensity in cells present near the scratch (white arrows) depicts increased immunoreactivity for TG2 that was attenuated in presence of Ca^2+^-chelator, BAPTA-AM. Scale bar = 100 μm. Fluorescence intensity in cells present near the scratch was measured using Image-Pro Plus software and plotted as fold change in IOD **(D)**. **(E)** Representative Western blots for TG2 in presence of a Ca^2+^-chelator, BAPTA-AM, showing that resveratrol-induced slower mobility TG2 form is sensitive to Ca^2+^ levels. Native proteins isolated from resveratrol treated SHY_TG2_ and Panc-28 cells in the presence or absence of BAPTA-AM (10 μM) from near the scratch were isolated, separated under non-denaturing conditions, and blotted with anti-TG2 antibody. The experiments were repeated three times.

Since intracellular Ca^2+^ levels determine TG2 conformational state [34], we hypothesize that the slower migratory form of TG2 may result from resveratrol-induced changes in intracellular calcium concentrations [35]. To test this possibility, we first performed immunofluorescence studies using a membrane permeable Ca^2+^ indicator dye, Fluo4-AM, in scratch assays. The fluorescence intensity in cells near the scratch was quantified as integrated optical density (IOD) by Image-Pro Plus software and plotted (Figure 6A and B). We found that resveratrol (1 μM or 10 μM for 48 h) significantly increased Ca^2+^ fluorescence intensities in SHY_TG2_ cells present near the scratch (Figure 6Af, g and h). In our experiments, we observed that along with migrating cells, some cells away from the scratch also exhibited increased calcium fluorescence. However, the response was more significant in the cells near the scratch. Changes in the cytoplasmic calcium levels are known to be transient and all the cells do not fluoresce at the same time. Therefore we did not get synchronized calcium labeling. The effect on the cells away from the scratch may be due to the secondary response of the cells near to scratch, also shown in some previous studies [36,37]. Similar increase in fluorescence intensity of Ca^2+^ was not observed when SHY_TG2_ cells were incubated with a Ca^2+^-chelator, BAPTA-AM (10 μM), in the presence of either concentration of resveratrol (1 or 10 μM) (Figure 6Ai and j). In contrast to SHY_TG2_ cells, we did not observe such resveratrol-induced increased Ca^2+^ fluorescence in SHY_vector_ cells (Figure 6Aa-e).

The increased levels of Ca^2+^ in resveratrol-treated migratory SHY_TG2_ cells prompted us to test whether changes in intracellular Ca^2+^ level are responsible for the increased TG2 immunoreactivity in resveratrol-treated SHY_TG2_ cells (as seen in Figure 2Ai and o). We further checked the immunofluorescence signal for TG2 in the presence of a Ca^2+^-chelator, BAPTA-AM (10 μM), in combination with resveratrol (1 or 10 μM, 48 h) in scratch assays. Our immunofluorescence data demonstrated that BAPTA-AM significantly attenuated the resveratrol-induced immunoreactivity of TG2 (red color, shown by arrow) and was similar to control-level in migratory cells (Figure 6C and D). To further confirm the Ca^2+^-induced changes in TG2 protein, we performed Western blots for TG2 under non-denaturing conditions with native proteins isolated from BAPTA-AM (10 μM) and resveratrol (1 or 10 μM, 48 h) treated SHY_TG2_ cells present near the scratch. The Western blot for TG2 in Figure 6E demonstrated that presence of BAPTA-AM reduces the intensity of the slower mobility form of TG2 in resveratrol-treated cells (compare lane 2, 3 and 5, 6). These results indicated the slower migratory form of TG2 which appears in the presence of resveratrol is sensitive to Ca^2+^ ions, and this form of TG2 may be responsible for the higher immunoreactivity of TG2 in cells present near the scratch.

## Discussion

The role of TG2 in cancer cell metastasis and drug resistance is well documented [11], however, its precise molecular mechanism in migration and invasion is not fully understood. Here we report that TG2 promotes migration and invasion of SH-SY5Y neuroblastoma cells. In contrast, we have previously shown that retinoic acid-induced TG2 promotes differentiation of SH-SY5Y cells [21]. In order to understand the opposite behavior of TG2 on cell migration and differentiation, we used a natural compound, resveratrol. Various in vivo studies in cancer models including neuroblastoma confirms the anticancer properties of resveratrol [24,25], however, in vitro studies have needed higher concentrations (>100 μM) of resveratrol to induce apoptosis in cancer cells. Moreover, such concentrations are not achievable in vivo. Hence we use physiologically relevant concentrations of resveratrol (1 μM and 10 μM) in our studies to assess its effects in cancer cells. Our experiments showed that resveratrol even at 1 μM concentration significantly inhibited migration/invasion of TG2 containing cells (SHY_TG2_ and pancreatic cancer cells) (Figure 1A-H). The observed inhibitory effects of resveratrol on migration was not due to the lower viability of cells, since the number of viable cells following DMSO and resveratrol treatment (1 μM or 10 μM) were almost similar in cell viability assays carried out by trypan-blue exclusion method (Additional file 1: Figure S2). We also observed that resveratrol significantly increased the TG2 immunoreactivity in migratory SHY_TG2_ and pancreatic cancer cells (Figure 2) without affecting TG2 protein level (Figure 4A). These results prompted us to investigate how resveratrol increases TG2 immunoreactivity without changing the protein levels in cells. The Western blots under non-denaturing conditions detects an additional TG2 protein band of slower mobility in total native proteins from resveratrol-treated SHY_TG2_ and Panc-28 cells present near the scratch (Figure 5A, lanes 2 and 3). Although both cellular fractions (cytoplasmic and plasma membrane) exhibit the slower mobility TG2 band, the membrane fractions exclusively has this TG2 form in resveratrol-treated SHY_TG2_ and Panc-28 cells present near the scratch (Figure 5B, lanes 2, 3, 5 and 6). The slow mobility band may be the open structure form of TG2 and the higher mobility band may be compact form of TG2, also visualized by others using native polyacrylamide gel electrophoresis [5,34,38].

Furthermore, we also observed a significant increase in fluorescence levels of Ca^2+^ in response to resveratrol in migratory SHY_TG2_ cells (Figure 6Ag and h). The reduced immunoreactivity of TG2 by depleting the intracellular Ca^2+^ with BAPTA-AM in resveratrol-treated SHY_TG2_ migrating cells (Figure 6Cb-e) correlate with the Ca^2+^ dependent changes in TG2 immunoreactivity. The observation that BAPTA-AM reduced the level of the slower mobility form of TG2 in resveratrol treated cells (Figure 6E, lanes 5 and 6) further support the requirement of Ca^2+^ ions in the structural modification of TG2. Resveratrol induced calcium release is supported by recent studies in myotubes and chromaffin cells [35,37]. Previous reports suggest the existence of two forms of TG2 depending on the Ca^2+^ ion concentrations in cells with a compact closed form at low Ca^2+^ concentration and an open extended form at higher Ca^2+^ concentration [34,39-44]. Hence the observed resveratrol-induced increased Ca^2+^ levels (Figure 6) sensitize TG2 which in turn converts from the compact form into the open extended structure (slow mobility form) in the cytoplasm, and this extended slow mobility form mobilizes to the plasma membrane. These observations suggest that the increased immunoreactivity of TG2 in resveratrol-treated migratory SHY_TG2_ and pancreatic cancer cells is due to greater antigen accessibility of the anti-TG2 antibody to the Ca^2+^-sensitized open low migratory form of TG2. This also explains the reduced levels of TG2 immunoreactivity in SHY_mutant_ cells (Figure 2) even in presence of similar levels of total TG2 protein as in SHY_TG2_ cells. Resveratrol-induced increased Ca^2+^ levels might fail to convert mutated TG2 into the open form, reducing antigen access of the anti-TG2 antibody in SHY_mutant_ cells.

Absence of the slow molecular form of TG2 after incubation of native total SHY_TG2_ proteins with resveratrol (1 or 10 μM) alone or in combination with Ca^2+^ in an in vitro cell-free system (Additional file 1: Figure S3) suggest that the conversion of closed to open structure form of TG2 is not due to a direct interaction between resveratrol and TG2, and that other factors might also be involved in resveratrol-induced TG2 conversion. We also tested the possible interaction between TG2 and resveratrol by computational modeling using different approaches. The COACH server and TM-ALIGN analysis failed to predict the significant interaction, however, another program BSP-SLIM (for low-resolution ligand-protein docking) showed some docking positions on TG2, but it was difficult to evaluate if they indeed interact (Additional file 1: Figure S4a and S4b). Thus the cell-free system and bioinformatics approaches concluded that resveratrol did not interact significantly with TG2. Our observation that the slower mobility TG2 form is non-phosphorylated and the higher mobility form is phosphorylated (Figure 5C) suggests that resveratrol induces dephosphorylation of the slower mobility TG2 form which travels from cytoplasm to plasma membrane. How shuttling of the Ca^2+^-sensitive open slow mobility dephosphorylated TG2 form to cytoplasmic side of plasma membrane occurs is not clear. A recent study revealed that a heat shock protein (Hsp70) mediates TG2 transport to the plasma membrane [45]. Our results that the open structure slow mobility TG2 band was present in both cytoplasmic and membrane fractions, and the higher mobility form was present only in cytoplasmic fractions (not in membrane fractions) in SHY_TG2_ and Panc-28 cells (Figure 5B) suggests that a chaperone-like activity of Hsp70 might help in Ca^2+^-induced TG2 conversion, and interaction of Hsp-70 to the open slow mobility TG2 form might facilitate the transport of this form of TG2 from cytoplasm to plasma membrane. Previous studies indicate that acetylation [46] and ubiquitination of TG2 [47] inhibit its transamidation activity and degrade the protein into smaller fragments. In our studies increased transamidation activity by resveratrol and absence of smaller proteolytic fragments of TG2 suggest that resveratrol does not induce the acetylation and ubiquitination of TG2.

In response to various stimuli, TG2 is either transported to the cytoplasmic or extra-cellular side of plasma membrane [48]. On the cytoplasmic side of the plasma membrane, TG2 activates phospholipase C (PLC) and RhoA-ROCK-2 signaling pathways [21,22,31], while on the cell surface, TG2 acts as a co-receptor for integrins to promote cell attachments [49]. TG2 is known to positively regulate the expression and activity of metalloproteinases (MMPs)-2 and −9 in cancer cells [10,50]. MMPs are a family of secreted proteins known to promote cancer cell migration through acting in extracellular environment [51]. In our scratch assays with SHY_TG2_ and Panc-28 cells, resveratrol at 1 μM or 10 μM concentrations did not affect TG2 localization to the extracellular membrane surface (Figure 3A and B) or its secretion into culture medium (ELISA, Additional file 1: Figure S1). Also we did not observe any effects on enzymatic activity of matrix metalloproteinases (MMPs)-2 and −9 in culture medium collected after resveratrol (1 or 10 μM) treatments in scratch assays (as revealed by gelatin zymography, Additional file 1: Figure S5) suggesting that intracellular (not extracellular) TG2-mediated signaling events are involved in the inhibitory effects of resveratrol in cell migration. Incubation with Sirtinol (an inhibitor of Sirt-1, a well-known substrate of resveratrol) did not attenuate the resveratrol-effects on migration of SHY_TG2_ and Panc-28 cells suggesting that the observed inhibition in cell migration by resveratrol is Sirt1-independent (Additional file 1: Figure S6) [52].

**Figure 7:**
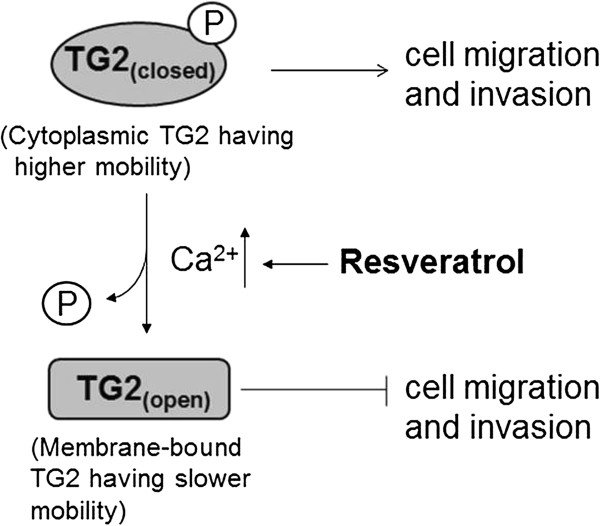
Putative model explaining the underlying mechanism of resveratrol effects on TG2-mediated cell migration.

By demonstrating that resveratrol induces TG2 translocation to the plasma membrane, and increases intracellular Ca^2+^ levels, we propose a putative model which explains the underlying mechanism of resveratrol effects on TG2-mediated cell migration (Figure 7). Under normal conditions, cytoplasmic TG2 remains in its phosphorylated compact form and induces cell migration and invasion. Its GTP-binding function plays a major role in mediating these effects and requires low intracellular calcium concentrations. Following resveratrol exposure increased intracellular Ca^2+^ levels converts closed TG2 form into the open extended form which in turn, probably with the help of Hsp70 protein, translocates to the cytoplasmic side of plasma membranes. On the plasma membrane, TG2 activates the PLC signaling pathway [31]. Activation of PLCδ pathways further amplify the Ca^2+^ levels in the cytoplasm by releasing Ca^2+^ from intracellular organelles, which in turn increases the transamidation activity of TG2 for the prevention of cell migration/invasion. The observations that transamidation activity of TG2 is required for cell migration is supported by less mobility of transamidation-defective SH-SY5Y cells (SHY_mutant_) in scratch assays (Figure 1A), and the reduced migration of SHY_TG2_ cells and Panc-28 cells in the presence of a TG2 transamidation inhibitor, monodensylcadevarine (Additional file 1: Figure S7). A recent report demonstrating the role of a Ca^2+^-binding protein, S100A4, in TG2-mediated migration further supports our observations of a Ca^2+^ requirement for TG2-mediated cancer cell migration [53].

## Conclusions

Together, the present study indicates that TG2 is one of the proteins involved in cancer cells migration/invasion, and supports its use as a therapeutic target in cancer prevention. Although higher concentrations of resveratrol (~100 μM) induces cell death in cancer cells [24], the current study suggest that lower physiologically relevant dose of resveratrol (~1 μM) prevent the migration and invasion of cancer cells from the primary site. Resveratrol has shown promising results for the treatment of many diseases using animal models [27] and recently findings have been extended to humans. For example, in a recent clinical trial, 30 days of resveratrol treatment (150 mg/day) induced beneficial metabolic changes in obese humans [54]. Rapid metabolism and poor bioavailability is generally thought to be a limiting factor in the use of resveratrol as a therapeutic agent in translational medicine. However, recent studies show that resveratrol sulfate, a metabolite of resveratrol is converted back into resveratrol inside the cells and the effective concentration of resveratrol inside the cells may be higher [55]. Since retinoic acid treatment increases TG2 protein level and resveratrol inhibits the migration of TG2-expressing cells, this study also proposes the use of resveratrol with retinoic acid for the treatment of neuroblatoma.

## Abbreviations

BAPTA-AM: 1,2-Bis (2-aminophenoxy) ethane-N, N, N’, N’-tetraacetic acid tetrakis (acetoxymethyl ester); DMSO: Dimethyl sulphoxide; DTT: Dithiothreitol; IOD: Integrated optical density; MDC: Monodensylcadavarine; PBS: Phosphate buffered saline; PMSF: Phenylmethylsulfonyl fluoride; res: Resveratrol; TG2: Tissue-transglutaminase.

## Competing interest

The authors declare that they have no competing interest.

## Authors’ contributions

AK and USS designed all experiments. AK performed experiments, KBW helped in Ca^2+^-related experiments, and JH conducted computational modeling. AK, HAL, DJD and USS analyzed the data and wrote the manuscript. All authors read and approved the final manuscript.

## Pre-publication history

The pre-publication history for this paper can be accessed here:

http://www.biomedcentral.com/1471-2407/14/256/prepub

## Supplementary Material

Additional file 1 Supplementary Data. Click here for file
