# Erratum to: Conformational changes and translocation of tissue-transglutaminase to the plasma membranes: role in cancer cell migration

**DOI:** 10.1186/s12885-016-2567-8

**Published:** 2016-08-08

**Authors:** Ambrish Kumar, Jianjun Hu, Holly A. LaVoie, Kenneth B. Walsh, Donald J. DiPette, Ugra S. Singh

**Affiliations:** 1 Department of Pathology, Microbiology and Immunology, School of Medicine, University of South Carolina, Columbia, SC 29209 USA; 2 Department of Computer Science and Engineering, University of South Carolina, Columbia, SC USA; 3 Department of Cell Biology and Anatomy, School of Medicine, University of South Carolina, Columbia, SC USA; 4 Department of Pharmacology, Physiology and Neuroscience, School of Medicine, University of South Carolina, Columbia, SC USA; 5 Department of Internal Medicine, School of Medicine, University of South Carolina, Columbia, SC USA

## Erratum

Unfortunately, the original version of this article [1] contained an error in Fig. 1. In Fig. 1A the images in column 2 row 1, column 3 row 1 and column 1 row 6 are duplicated in column 1 row 5, column 3 row 5 and column 3 row 6, respectively. This duplication does not affect the results, findings, interpretation, conclusions, or the scientific basis of the article. The graph and statistical analysis related to the photomicrograph in Fig. 1A are correct and taken from the correct images. The correct version of Fig. 1 is below.

**Fig. 1:**
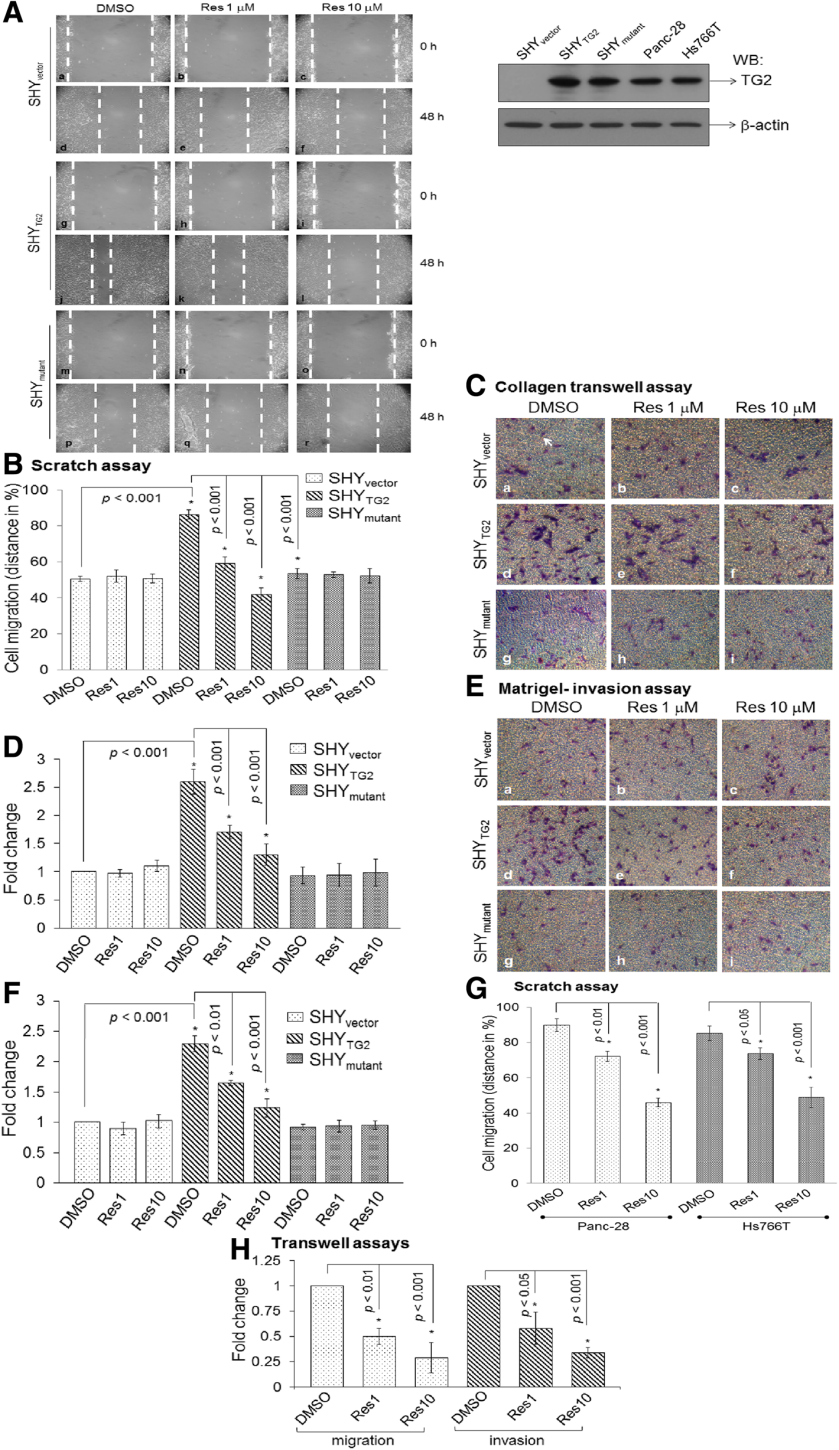
Cell migration and invasion assays. **A** Representative images from the scratch assays showing the migration of SHY_vector_, SHY_TG2_, and SHY_mutant_ cells. Mytomycin C treated cells were preincubated with resveratrol (1 μM and 10 μM) for 24 h; a scratch was made and further incubated with resveratrol. At 0 h and 48 h after scratch, cells were photographed. Dotted lines represent the edge of migrated cells in the scratch. Upper right panel: Western blot for TG2 protein using 10 μg total cell extract from SHY_vector_, SHY_TG2_, SHY_mutant_, Panc-28, and Hs766T cells. **B** The distance covered by cells in the original empty area was measured and plotted in %. Bars represent mean ± SD of three independent experiments. *p value < 0.05. **C-E** Images from collagen-transwell and matrigel-transwell assays. After 48 h with or without resveratrol treatment, cells were trypsinized and seeded on collagen-transwell (**C**) or matrigel-transwell inserts in the presence of resveratrol (**E**). After 15 h, migrated cells on the lower side of inserts were stained with Hema-3 stain (arrow), counted from ten random fields and plotted (**D** and **F**). Bars are mean ± SD of at least three independent experiments and *p value < 0.05. **G** Bar diagram represents the migration of Panc-28 and Hs766T cells in scratch assays in the presence of resveratrol as performed with SH-SY5Y cells. Migrated cells into the original empty area were photographed and plotted. Bars are mean ± SD of three independent experiments. *p value < 0.05. **H** Migration and invasion assays for Panc-28 cells were carried out as with neuroblastoma cells in transwell inserts. Migrated/invaded cells were counted from 10 random fields and plotted. Bars are mean ± SD of three independent experiments. *p value < 0.05

As Dr. Ugra Singh is no longer at the University of South Carolina, the corresponding author has been changed to Dr Donald J DiPette (Donald.dipette@uscmed.sc.edu) in the author details above.
