# Supplementary material for: Conformational changes and translocation of tissue-transglutaminase to the plasma membranes: role in cancer cell migration
Source: BMC Cancer. 2014 Apr 11;14:256. doi: 10.1186/1471-2407-14-256 (PMC4021189; doi:10.1186/1471-2407-14-256)
Supplement: Additional file 1 — Supplementary Data. [file 1471-2407-14-256-S1.doc]

Conformational changes and translocation of tissue-transglutaminase to the plasma membranes: role in cancer cell migration

Ambrish Kumar^1^, Jianjun Hu^2^, Holly A. LaVoie^3^, Kenneth B. Walsh^4^, Donald J. DiPette^5^, Ugra S. Singh^1^

^1^Department of Pathology, Microbiology and Immunology, School of Medicine, University of South Carolina, Columbia, SC, USA.

^2^Department of Computer Science and Engineering, University of South Carolina, Columbia, SC, USA.

^3^Department of Cell Biology and Anatomy, School of Medicine, University of South Carolina, Columbia, SC, USA.

^4^Department of Pharmacology, Physiology and Neuroscience, School of Medicine, University of South Carolina, Columbia, SC, USA.

^5^Department of Internal Medicine, School of Medicine, University of South Carolina, Columbia, SC, USA.

**Supplementary Data**

**Supplementary Figure S1**

**
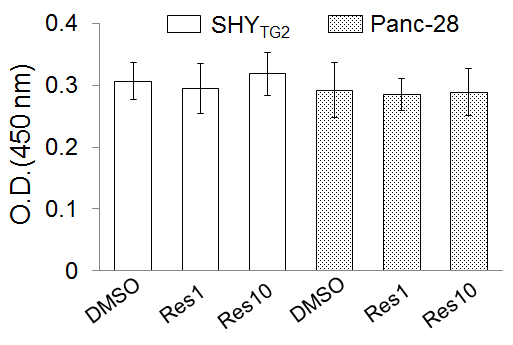
**

**Supplementary Figure S1.** Enzyme-linked immunosorbent assay (ELISA) to detect secreted TG2 in culture medium. Scratch assays with SHY_TG2_ and Panc-28 in presence of resveratrol (1 or 10 μM, 48 h) were performed as mentioned in material and methods sections. 150 μl of culture medium in 150 μl of carbonate buffer (50 mM, pH 9.6) was used to coat 96 well ELISA plate for overnight at 4ºC. After washing with 1x PBS, wells were blocked overnight at 4ºC with 5% nonfat dry milk/PBST followed by incubation with anti-TG2 antibody (1:500 dilution in 2.5% nonfat dry milk/PBST) for overnight at 4ºC. After washing with PBS, anti-mouse HRP-conjugated secondary antibodies (1:5000 dilution in 2.5% nonfat dry milk/PBST, Santa Cruz) was added, and incubated for 2 h. The signals were detected by adding tetramethylbenzidine (TMB) substrate (100 μl) into each well for 15 min. Optical density was measured at 450 nm after adding stop solution (50 μl) and plotted.

**Supplementary Figure S2**

**Supplementary Figure S2.** Trypan-blue cell viability assay. Representative bar diagram represents the viability of cells after resveratrol treatment. Neuroblastoma and pancreatic cancer cells (Panc-28 and Hs766T cells) were treated with resveratrol (1 μM or 10 μM) for 4 days in complete culture medium (with 10% FBS). Cells treated with equal volume of DMSO were used as control. After treatments, cells were trypsinized with trypsin-EDTA, counted by trypan-blue exclusion method (Sigma-Aldrich, St. Louis, MO) and plotted. DMSO-treated cell was considered as 100% and *p* < 0.05 was considered significant.

**Supplementary Figure S3**

**Supplementary Figure S3.** TG2-resveratrol interaction in *in vitro* cell-free system. In a test tube, native proteins (5 μg) isolated from SHY_TG2_ (upper panel) and Panc-28 cells (lower panel) present near the scratch in scratch assays were incubated with 1 μM or 10 μM of resveratrol alone or in combination with calcium chloride (0.1 or 1 mM) for 30 min. Reaction mixtures were mixed with 5x gel loading dye (without SDS and reducing agent), separated on 12% native gel and Western blots with anti-TG2 antibody were performed. Anti-TG2 antibody detects only one TG2 band of higher mobility, not the slower mobility form, in resveratrol containing samples (lanes 2 and 3). Addition of Ca^2+^ did not affect resveratrol interactions with TG2 (lanes 6-9). Although TG2 band with lower intensity was observed in samples containing 10 μM resveratrol and 1 mM CaCl_2_ (lane 9), this may be the due to proteolysis of TG2 in the presence of activated Ca^2+^-dependent proteinase in cell preparations. These data suggest that resveratrol is not directly interacting with TG2 to induce conformational changes.

**Supplementary Figure S4a**

Resveratrol

TG2: domain2

**Supplementary Figure S4b**

Resveratrol

TG2: domain2

**Supplementary Figure S4.** Prediction of TG2-resveratrol interaction through computational modeling. We submitted all four domain of TG2 to the COACH server, a meta-server for protein-ligand binding site prediction. COACH server predicted two potential ligand binding sites- one on domain 2 with a high confidence score of 0.72, and another on domain 4 with a high confidence score of 0.27. However, resveratrol was not included in the list of predicted ligand in COACH server.

To further investigate if TG2 interacts with resveratrol, we applied structural alignment of domain 2 of TG2 with all the 13 known proteins that interact with resveratrol ligand as annotated in protein data bank (PDB) database. All the alignment scores as aligned by TM-ALIGN are not significant to claim their structural similarity. Finally, we applied BSP-SLIM for low-resolution ligand-protein docking with TG2 protein with the resveratrol ligand. The results just show some docking positions, but difficult to evaluate if they indeed interact (supplementary figure 4a and b). Thus we concluded that resveratrol could not interact significantly with TG2.

**Supplementary Figure S5**

**Supplementary Figure S5.** Gelatin zymograms showing enzymatic activity of matrix metalloproteinase (MMP)-2 and -9 in response to resveratrol. Scratch assays in presence of resveratrol (1 or 10 μM) was performed as described in previous sections. After 48 h scratch, culture medium was collected, centrifuged at 12,000 g for 10 min to remove any residual cells, 10 μl medium was mixed with 5x loading dye and separated on 7.5% SDS-polyacrylamide gel containing 0.1% gelatin. Gels were washed with a wash buffer (50 mM Tris, pH 7.4, 5 mM CaCl_2_, 1 μM ZnCl_2_ and 2.5% Triton X-100) for 2 h to remove SDS and renature proteinases, incubated overnight with wash buffer (without Triton X-100), and stained with Coomassie G250 for 2 h at room temperature. Gels were de-stained with 20% methanol/4% acetic acid and photographed in Bio-Rad gel doc system.

**Supplementary Figure S6**

**Supplementary Figure S6.** Scratch assays in presence of Sirt1 inhibitor, sirtinol, and resveratrol. Representative bar diagram represents the migration of SHY_vector_, SHY_TG2_, and Panc-28 cells in presence of sirtinol in scratch assays. The scratch assays were performed as described in material and methods section in presence of 10 μM concentration of sirtinol (Sigma) alone or in combination with resveratrol (10 μM). Cell migration was monitored, and after 48 h, cells near the scratch were photographed. Cell migration in empty area was calculated in % and plotted. Experiments were repeated for three times and *p* < 0.05 was considered significant.

**Supplementary Figure S7**

**Supplementary Figure S7.** Scratch assays in the presence of 10 or 20 μM monodensylcadavarine (MDC), an inhibitor of transamidation activity, was performed with SHY_TG2_ and Panc-28 cells. After 72 h treatment, cells were photographed; distance covered by cells in the empty area was measured and plotted (**A and B**). Bars are mean ± SD of three independent experiments. **p* value < 0.05. Dotted lines represent the edge of the original scratch. After end of experiment, cells were trypsinized and cell viability was determined by trypan blue exclusion method (Sigma). Number of live cells was plotted as fold change (**C**). These data indicated that MDC inhibits the migration of TG2 expressing cells without affecting cell viability.
